# African Edibles

**Published:** 1858-03

**Authors:** 


AFRICAN EDIBLES.—In Barth's very instructive if not entertaining "*Travels and Discoveries in North and Central Africa*," we meet with frequent notices of the articles of food, chiefly from the vegetable kingdom, used by the inhabitants of those extensive regions. The subject is interesting, both in a hygienic and economic point of view; and as such we now bring it before our readers, without any regard to methodical arrangement, but rather as we find it in the volumes before us. Barth's starting point was Tripoli. He made an excursion, however, previously, in an eastern direction, through the Regency.

Soon after setting out, the travellers emerged from the palm-groves which constitute the charm of Tripoli. Then they came to the fine date-plantations of Zenzer, celebrated in the fourteenth century as one of the fairest districts of Barbary, and they pass by a great magazine of grain. Fine olive trees pleasingly alternate with the palm-grove, while the borders of the broad sandy paths were neatly fenced with the *cactus opuntia*, or prickly pair. As preparation for their nomadic life in crossing the desert, they laid in a supply of corn and dates. The fruit called gatuf of the batim tree, (*Pistacia Atlantica*,) or Barbary Mastich, is used by the Arabs for a variety of purposes. In other countries its fragrant and astringent resin is best known. The rearing of fruit trees seems to be a favorite occupation of the Berber race, even in the more favored spots of the Great Desert. The cultivation of the olive extends to the borders of the Desert. Saffron and olives are the two staple articles of industry in this region, the maritime one of Tripoli. The cultivation of grain is made productive by means of irrigation.

On the second departure of the travellers from Tripoli, their course was nearly due south, to Murzuk, on the border of the Desert. On the oases of Mizda, and some others still farther south, barley and wheat in cultivation were found in the vicinity of olive and fig trees. The soil around Mursuk, a little to the south of 21° N. latitude, is very arid: even in the plantations which surround it there are only a few favored spots, where, under the protection of a deeper shade of the date trees, a few fruit trees can be cultivated, such as pomegranates, figs, and peaches. With great labor, wheat, barley, gedheb, (or rather kédheb,) are cultivated. Culinary vegetables, including onions, are extremely scarce; milk, except a little from the goats, is quite out of the question.

In the oases of Ghat and Barakat, Guinea corn, gero or millet, (*Pennisetum typhordeum*,) is cultivated to a much greater extent than wheat or barley. Palm-groves are repeatedly passed, and irrigation witnessed in the fields and gardens. In the valley Nghakeli, richly overgrown with luxuriant herbage and adorned with fine tallha trees, was exhibited the first specimen of the *Balanites Œgyptiaca*, (hajilig, of the Arabs,) of which we shall soon speak. In this region, at the bottom of a valley skirting mountain masses, was seen the grass *Avena Forskalii*, which is very much liked by the camels. Here, also, the travellers partook of the flesh of the Wadán, (*Ovis trugdaphos*,) an animal very common in the mountainous districts of the Desert, and found often in company with the wild ox.

Though not ranking with edibles, we may mention, by the way, that in latitude 20° N., the senna plant (*Cassia senna*) appeared in tolerable quantity. More germain to our actual theme is the appearance, in this district, the valley of Gebi, of the absiga, (*Capparis Sodata*,) a variety of the caper, called siwak, or lirak, by the Arabs. This, writes Barth, "is an important bush, the fruit of which is not only eaten fresh, but also dried, and laid up in store; while the root affords that excellent remedy for the teeth which the Mohammedans, in imitation of their prophet, use to a great extent. The root, however, at least on the shores of the Tsad, by the process of burning, affords a substitute for salt. It is the most characteristic bush or tree of the whole region or transition between the desert and the fertile regions of Central Africa, between the twentieth and the fifteenth degrees of northern latitude.' In the course of his travels, Barth saw it nowhere of such size as on the northern bank of the Isa or Niger, between Timbuktu and Ghago; the whole ground which this once splendid and rich capital of the Singhay Empire occupied being at present covered and marked out by this celebrated bush. The berries, although only ripening, (August 22d,) afforded a slight but refreshing addition to the food of the travellers.

Skirting the mountain group of Tintellust, elevated 5000 feet above the ocean, Barth and his companions passed through the valley of Selufiet, in lat. 19° N., rich in trees and bushes, but without herbage. Here he met with his old acquaintance from the Said and Nubia, the dum-tree or dum palm, (*Cucifera Nigritia.*) This tree has a wide geographical range through Central Africa; but its chief region is that of Bornu proper. Its fruit is an essential condiment to the soup made of Negro millet or Guinea corn. At Tintellust, in the mountainous country of Air or Asben, which Barth calls the Switzerland of the Desert, he and his friends received a small supply of millet, butter, and a little fresh cheese, and they purchased two or three goats and a camel-load of durra, (*holcus sorghum,*) of the dietetic value of which last grain we shall speak hereafter.

Leaving his companions at Tintellust, Barth made a journey to Agades, in a southwesterly direction. On the way, in the village of Eghellal, at the foot of a mountain of the same name, his eyes were greeted with the sight of well-fed cattle returning from their pasture-grounds. "They were fine sturdy bullocks of moderate size, all with the hump, and of a glossy dark-brown color." It conflicts much with our ideas of the great desert of Sahara, to be told, as we are by our traveller, that in the valley of Aideras, in Air, he saw not only millet, but even wheat, the vine, and dates, and almost every species of vegetable. A little farther south, in the valley Budde, where the mimosas attained a large growth, Barth first became acquainted with the karengia or *Pennisetum distichum,* on the seed of which many of the Tawarek from Bornu, as far as Timbuktu, subsist more or less. The drink made from it is certainly not bad, resembling in coolness the fura or ghussub-water. The grass itself is a most nourishing food for cattle. The little burr-like seeds are, however, a great annoyance to the traveller in Central Africa, by attaching themselves to every part of the dress. Hence the necessity, not neglected even by the natives, to be always provided with small pincers, in order to draw out from the fingers the little stings which, if left in the skin, will cause sores.

As the market of a place affords a pretty good indication of the dietetic usages of the people, we may begin references of this nature by speaking of that of Agades. Negro millet is the real standard of the market, and in it the merchants of the town chiefly trade. The display of vegetables was poor; only cucumbers and molukhia (*corchorius olitorius*) being procurable in considerable plenty. The butchers' market was well furnished. Barth was gratified by a present, from a blacksmith friend of the place, with a large batta or leather box holding butter and vegetables, (chiefly melons and cucumbers,) and the promise of another sheep. In the more favored valleys of Air or Asben there are considerable herds of cattle. A long desert plateau intervenes between Asben and the Tigama country, a region the border one of the desert, and rich in cattle. Their slaves are busy in collecting and pounding the seeds of the karengia or azak, the *Pennisetum distichum*, which constitutes a great part of their food. The desert region just mentioned is the home of the giraffe, wild ox, ostrich, etc. In this part of the journey the travellers made the acquaintance of another tree, a native of Middle Sudan, named magaria by the Arabs, and kusubu by the Kanuri. It produces a fruit of a light-brown color, nearly equal in size to a small cherry, but in other respects more nearly resembling the fruit of the cornel, (*Cornus.*) When dried, it is pounded and formed into little cakes, which are sold all over Hausa as *tuwo-n-magaria*. It may be safely eaten by a European to allay his hunger for a while, till he can obtain something more substantial. The next district to the south, traversed by the line of the fifteenth degree of north latitude, is Damerghu, an undulating rich country, the granary of, and tributary to Asben. The production of grain consists in millet of the white species: durra or sorghum is not seen. Here the travellers met once again with the first poor specimens of the magnificent tamarind tree, the great ornament of Negroland.

The desert being passed, and the travellers fairly in Bornu, and of course in Central Africa, the dietary of the people, resulting from a richer soil, extensive pastoral regions, and rivers, became more abundant and varied. The change was more marked at Tasawa, where Dr. Barth made some stay. Before reaching this place, and near a village called Baibay, the varavan was surrounded by a great many women who offered for sale "godjia" or ground-nuts, and "dukkwa" or a sort of dry paste made of pounded Guinea corn, (*Pennisetum*,) with dates and an enormous quantity of pepper. This is the meaning of dukkwa in these districts; it is, however, elsewhere used as a general term signifying only paste, and it is often employed to denote a very palatable sort of sweetmeat made of pounded rice, butter, and honey. Leaving our readers to the indulgence of the sweet memory of such a compound, we shall not task their attention further at this time; but shall avail ourselves of a future opportunity to make them acquainted with the edibles and condiments of Negroland proper.

